# Distribution and Abundance of Glucocorticoid and Mineralocorticoid Receptors throughout the Brain of the Great Tit (*Parus major*)

**DOI:** 10.1371/journal.pone.0148516

**Published:** 2016-02-11

**Authors:** Rebecca A. Senft, Simone L. Meddle, Alexander T. Baugh

**Affiliations:** 1 Department of Biology, Swarthmore College, Swarthmore, Pennsylvania, United States of America; 2 The Roslin Institute, The Royal (Dick) School of Veterinary Studies, The University of Edinburgh, Easter Bush, Midlothian, EH25 9RG, United Kingdom; Claremont Colleges, UNITED STATES

## Abstract

The glucocorticoid stress response, regulated by the hypothalamic-pituitary-adrenal (HPA) axis, enables individuals to cope with stressors through transcriptional effects in cells expressing the appropriate receptors. The two receptors that bind glucocorticoids—the mineralocorticoid receptor (MR) and glucocorticoid receptor (GR)—are present in a variety of vertebrate tissues, but their expression in the brain is especially important. Neural receptor patterns have the potential to integrate multiple behavioral and physiological traits simultaneously, including self-regulation of glucocorticoid secretion through negative feedback processes. In the present work, we quantified the expression of GR and MR mRNA throughout the brain of a female great tit (*Parus major*), creating a distribution map encompassing 48 regions. This map, the first of its kind for *P*. *major*, demonstrated a widespread but not ubiquitous distribution of both receptor types. In the paraventricular nucleus of the hypothalamus (PVN) and the hippocampus (HP)—the two brain regions that we sampled from a total of 25 birds, we found high GR mRNA expression in the former and, unexpectedly, low MR mRNA in the latter. We examined the covariation of MR and GR levels in these two regions and found a strong, positive relationship between MR in the PVN and MR in the HP and a similar trend for GR across these two regions. This correlation supports the idea that hormone pleiotropy may constrain an individual’s behavioral and physiological phenotype. In the female song system, we found moderate GR in hyperstriatum ventrale, pars caudalis (HVC), and moderate MR in robust nucleus of the arcopallium (RA). Understanding intra- and interspecific patterns of glucocorticoid receptor expression can inform us about the behavioral processes (e.g. song learning) that may be sensitive to stress and stimulate future hypotheses concerning the relationships between receptor expression, circulating hormone concentrations and performance traits under selection, including behavior.

## Introduction

The acute glucocorticoid stress response, regulated by the hypothalamic-pituitary-adrenal (HPA) axis, enables individuals to cope with challenges encountered in everyday life. The HPA axis facilitates responses through various metabolic changes, including mobilization of glucose to muscles and suppressing long-term processes such as reproduction and growth [1]. These metabolic changes are accomplished through glucocorticoid receptor activation. Although there are both intracellular and membrane-bound receptors, most studies have focused on the role of intracellular mineralocorticoid receptors (MR) and glucocorticoid receptors (GR).

Differential activation of these two receptors among the many tissues expressing them provides modular regulatory control over metabolic processes by altering transcription in the target cells [1,2]. Glucocorticoids (GCs; cortisol in humans, many mammals, and fish; corticosterone in rodents, birds, amphibians; [3]) are the primary endocrine product of the HPA axis and are secreted by the adrenal cortices into general circulation before crossing the cell membrane and binding to the hormone-binding domains of MR and GR receptors in target cells. The receptor-ligand complex then enters the nucleus, homodimerizes, binds to hormone response elements specific to the receptor’s DNA-binding domain, and initiates transcription of target genes. The complete network of genes whose transcription is dependent on GR or MR activation is largely unknown, though a recent study examining GR target genes in mouse liver has identified a large regulatory map with over 300 GR-bound promoters, including genes for other transcriptional regulators and metabolic enzymes [4]. In addition, activity of MR and GR regulates the negative feedback processes of the axis itself, principally by binding to GR in the hypothalamus and pituitary, thereby inhibiting the secretagogues that lead to further elevations in glucocorticoid concentrations [5–7]. The diverse regulatory network for GR and MR, which is tissue and cell type dependent, is consistent with the wide variety of effects triggered by glucocorticoid release [1]. To better understand this complex regulatory system, an understanding of the distribution and abundance of these receptors across various tissues and cells is necessary. Moreover, because of its upstream location in the HPA axis, receptor expression in the brain has the potential to explain variation, intra- and inter-specifically, in stress physiology. In particular, the hypothalamus and hippocampus (hereafter HP) are two key regions because of their involvement in HPA regulation and roles in mediating diverse behaviors [3].

A handful of published distribution maps have documented MR and GR expression through *in situ* hybridization in a few species [8–14]. In songbirds, however, these maps are relatively limited in scale and resolution, exploring only a few regions and largely reporting only presence or absence of MR or GR or both. The mineralocorticoid receptor is reported to be localized to several discrete regions, most notably in HP [8–10]. In one study, MR was abundant in song-related regions such as the lateral part of the magnocellular nucleus of the anterior nidopallium (LMAN) and the robust nucleus of the arcopallium (RA) [14]. Glucocorticoid receptors are known to be more widely distributed, and are known to be abundant in the cerebellum, HP and paraventricular nucleus of the hypothalamus (hereafter PVN; [8–10]).

The great tit (*Parus major*) has served as an important model organism in behavioral ecology, particularly in animal personality research [15–17] and animal communication [18–20]. The HPA axis is known to modulate behavior in songbirds generally (e.g. locomotor [21]; vocal behavior [22]) and thought to contribute to the mechanistic basis of animal personality in this species specifically [23–25]). Further development of this species as a model for mechanistic studies of natural behavior could be facilitated by a better understanding of key neuroendocrine traits, including stress hormone receptors. Toward that aim, we quantified MR and GR abundances across 48 regions of interest, including several major song system nuclei (e.g. RA, LMAN, hyperstriatum ventrale, pars caudalis [HVC]).

Although a description of the neural distribution and abundance of MR and GR is itself a useful contribution towards generating hypotheses concerning the behavioral effects of glucocorticoids, it is also of interest as a means of understanding how a hormone may coordinate activity across different regions of the brain. The simultaneous and diverse effects of steroid hormones may connect traits in an individual’s phenotype, liberating or constraining their independence evolution, an idea referred to as hormonal pleiotropy [24–29]. Here we make an initial foray into this question by testing how the expression of MR and GR in two regions, the HP and PVN, covary across a sample of 25 individuals. This line of work, especially if expanded to other tissues throughout the body [30], has the potential to illuminate neuroendocrine mechanisms of phenotypic integration and constraint [24,25,31,32].

## Methods

### Animals

Protocols used in the present study were approved under permit 35–9185.81/G-10/76 by District administration Freiburg Department of Agriculture, Rural areas, Veterinary and Food Administration, Baden-Wuerttemberg, Germany at the Max Planck Inst. Ornithology, Radolfzell to Michaela Hau and Alexander Baugh. One female great tit (*Parus major*) was used to develop the MR/GR distribution map. This bird was one member of a cohort of twenty-eight great tits collected from a nestbox population in 2009 in Westerheide, NL and held in captivity at the Netherlands Institute for Ecology (Heteren, NL) after reaching nutritional independence. In November 2010, the birds were transported by van to the Max Planck Institute for Ornithology (Radolfzell, Germany). After two weeks of quarantine, they housed singly in outdoor aviaries (3 x 3 x 2 meters high) in alternating male-female adjacencies. This captivity facilitated control of the social environment (birds were housed singly) and the nutritional environment (a diet that including egg, mealworms, fresh vegetables and a custom bird food mixture was provided ad libitum). In total, 28 birds were collected for this study, but after the death of two and a tumor developed in the third, the sample size shrunk to 25 birds. The distribution map was created from one female and expression of GR and MR in HP and PVN was explored across 25 birds.

### In situ hybridization

In December 2012 we collected the brain tissue. In less than three minutes from entering the aviary, each bird was captured by hand net and brains were removed by rapid decapitation and frozen on a sheet of sterile aluminum foil in direct contact with a block of dry ice. Brain tissue was transported on dry ice to the Roslin Institute at the University of Edinburgh and mounted with OCT (TissueTek). We completely sectioned the brain coronally with a cryostat (Bright OTF/AS), from caudal to rostral at 15 μm thickness. Sections were thaw-mounted onto polysine pretreated slides (four sections per slide), which were stored at -80°C. We chose a total of 14 slides from the individual creating the basis for the distribution map (7 GR, 7 MR, in pairs distributed throughout the brain) for *in situ* hybridization. In addition, four slides (2 GR, 2 MR) were process for each of the 24 remaining birds in the cohort.

The protocol used here was previously described [9,10]. Briefly, selected slides were brought to room temperature in sealed slide boxes, fixed with paraformaldehyde, rinsed with PBS and treated with a series of increasingly concentrated ethanol to dehydrate the tissue. After air drying, slides were incubated for two hours at 50°C in a prehybridization solution. Following prehybridization, slides were incubated overnight (16–18 h) with S^35^-labeled radioactive sense or antisense riboprobes for zebra finch GR or MR [9,10,33] at 55°C. These probes have proven successful with other songbird species including the white-crowned sparrow [33]. Slides were then washed three times in saline-sodium citrate (SSC) for 5 minutes each and subsequently incubated with RNAse-A for one hour at 37°C. This was followed by SSC washes and ethanol dehydration. Slides were then dipped in autoradiographic emulsion and air dried and left to expose in sealed boxes at 4°C for 5 weeks before developing and counterstaining with haematoxylin and eosin. Finally, slides were dehydrated and coverslipped with DPX mountant (Sigma, St. Louis, MO, USA). Slides incubated with sense probes showed no detectable hybridization signal.

### Image Collection

One MR distribution map slide had an excessive amount of silver grain expression from a mistake when coverslipping and thus was not photographed or included in subsequent analyses. For each section, we identified regions of interest (ROIs) using the Stereotaxic Canary Atlas and Zebra Finch Atlas [34,35]. We photographed one frame near the center of these ROIs under transmitted bright-field illumination (Zeiss Akioskop and SPOT camera software) at 40X magnification [9]. Additionally, in order to account for nonspecific binding, one off-tissue background frame was captured for each ROI by panning the field of view to the left or right until off tissue [12]. For the distribution map, we took one background image per section. In photographing, light levels were consistent across all samples such that silver grains achieved maximum contrast with the background tissue. We also consistently used the auto-exposure setting.

### Quantification

**Fig 1:**
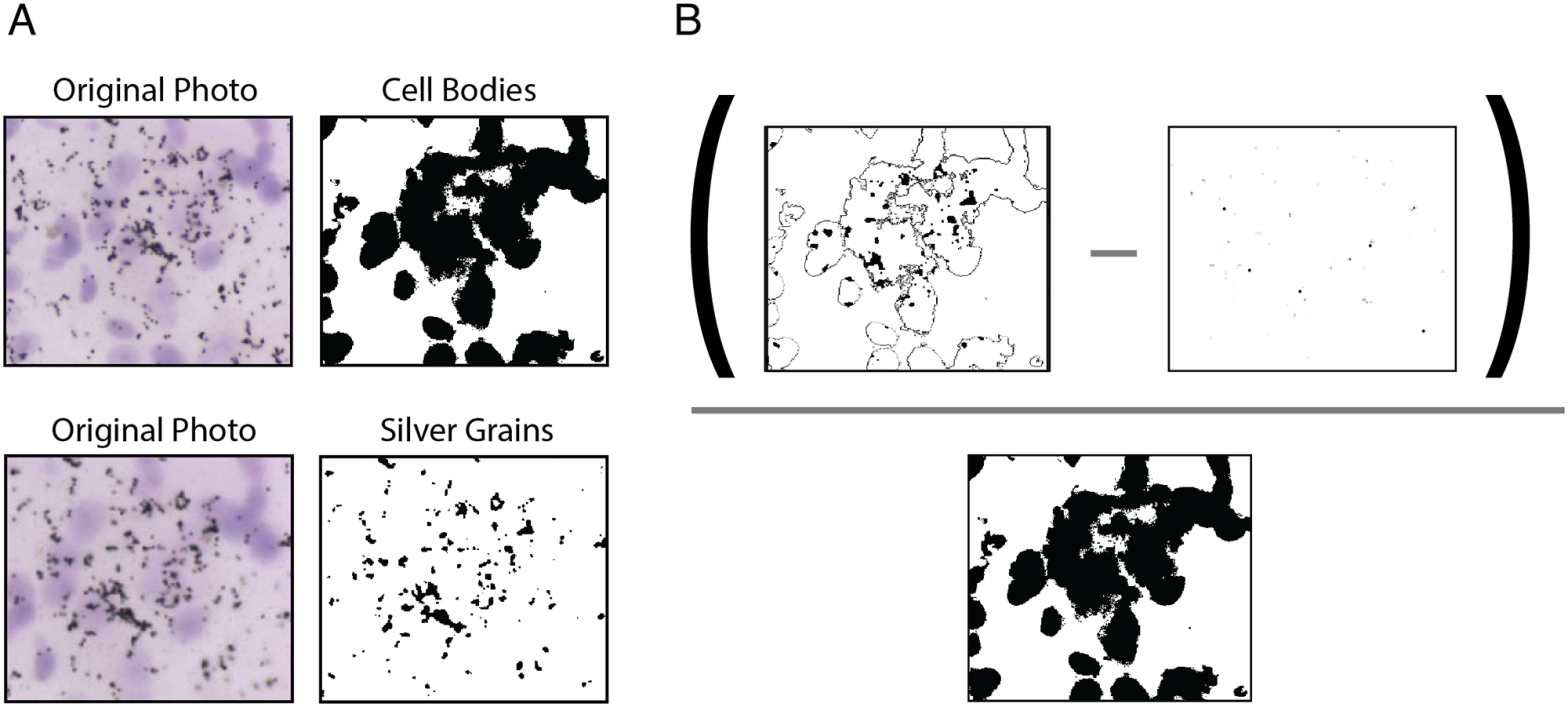
Image segmentation in Image J. A custom macro segmented images of silver grains over cell bodies into separate cell body (A, top) and silver grain (A, bottom) binary images. Expression was calculated as the pixels of silver grains superimposed over cell bodies subtracting a background level of silver grains all divided by the cell body area (B). This allowed us to control for variation in the number of cell bodies present in different regions.

Images were analyzed for silver grain expression in ImageJ (NIH). A custom macro utilized image segmentation to measure area (number of pixels) of silver grains superimposed over cell bodies (Fig 1). From this area, the macro subtracted area of silver grains from a background, off-tissue image in order to control for non-specific binding [12]. This area was then divided by the area of cell bodies, as number of cells differed across regions. The resultant measure is one of relative silver grain expression: area of silver grain expression over cells not attributable to nonspecific binding divided by the total area of cell bodies.

We binned these values into four categories: zero, low, medium, and high expression. We divided the range of positive silver grain densities into three equal partitions with the fourth category representing expression at or below background levels. This method does not directly compare MR and GR expression to each other, as differences in probe sequence and binding efficacy may affect the precise relationship between silver grains present and actual number of mRNA molecules [36]. Thus we binned mRNA levels for each receptor based on the range of values for that receptor. Finally, the high category was assigned to regions with greater SG than the maximum of the medium range (max expression for any given area was 0.1088 in MR and 0.1503 in GR). Expression level is indicated in the map by size of dot (larger corresponding to higher levels of mRNA).

As a framework for our distribution map, we followed the basic layout used in the Canary Atlas [34]. Using Adobe Illustrator (CS5) ^®^ we custom illustrated the layout of each plate based on our counterstained great tit tissue sections. All representative tissue images for the distribution map were adjusted for contrast, brightness, and hue in Adobe Photoshop (CS5).

## Results

**Fig 2:**
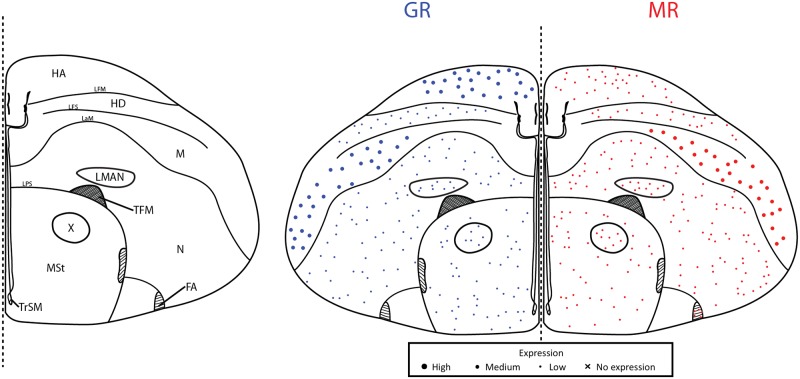
Plate 6 (A4.23–4.41 in the zebra finch atlas). Legend (left) and map (right). GR (blue, left) and MR (red, right) expression is indicated by dot size. An “X” indicates that the background expression met or exceeded the expression in the region. Area abbreviations can be found in Table 2.

**Fig 3:**
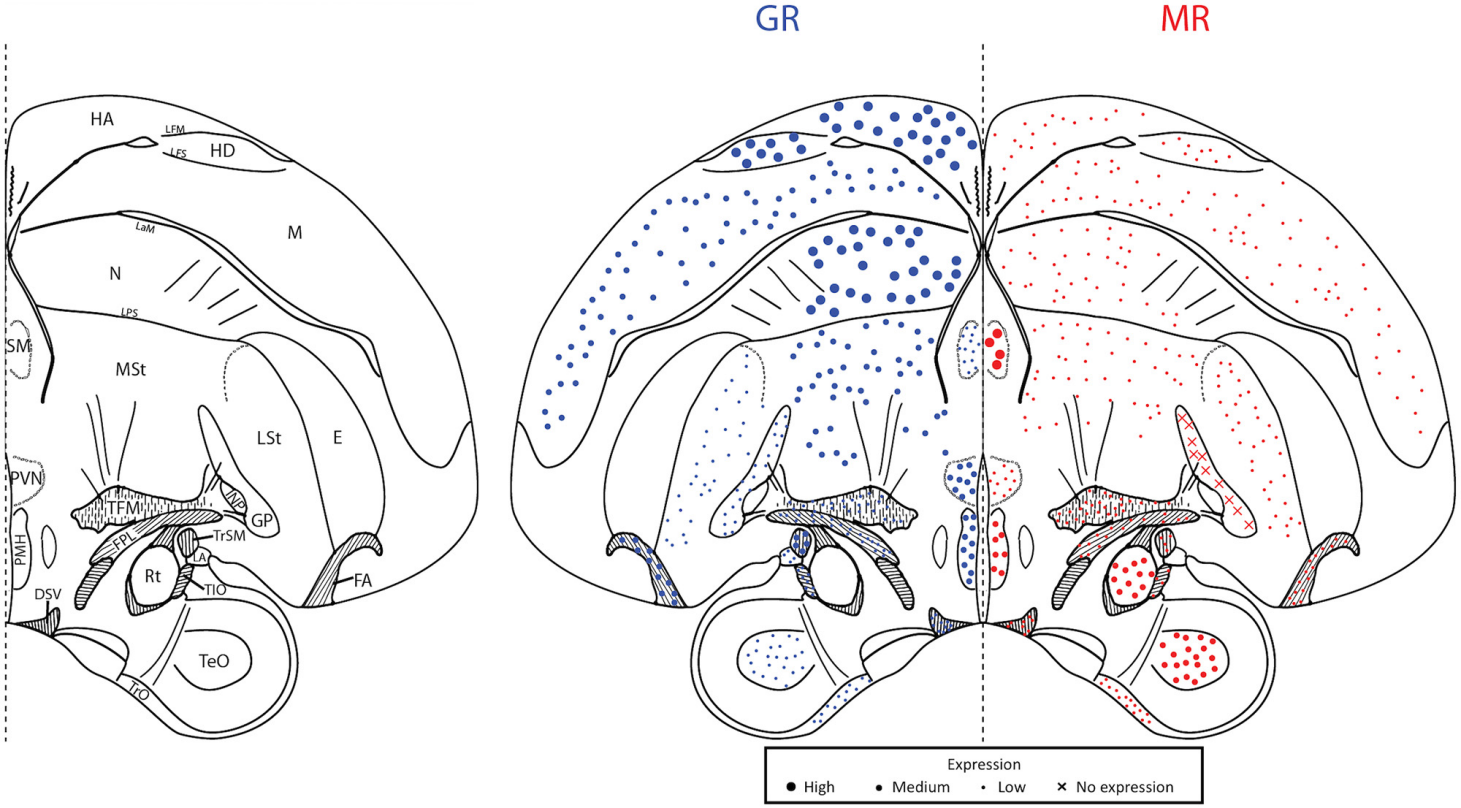
Plate 12 (~A2.97 in the zebra finch atlas). Legend (left) and map (right). GR (blue, left) and MR (red, right) expression is indicated by dot size. An “X” indicates that the background expression met or exceeded the expression in the region. Area abbreviations can be found in Table 2.

**Fig 4:**
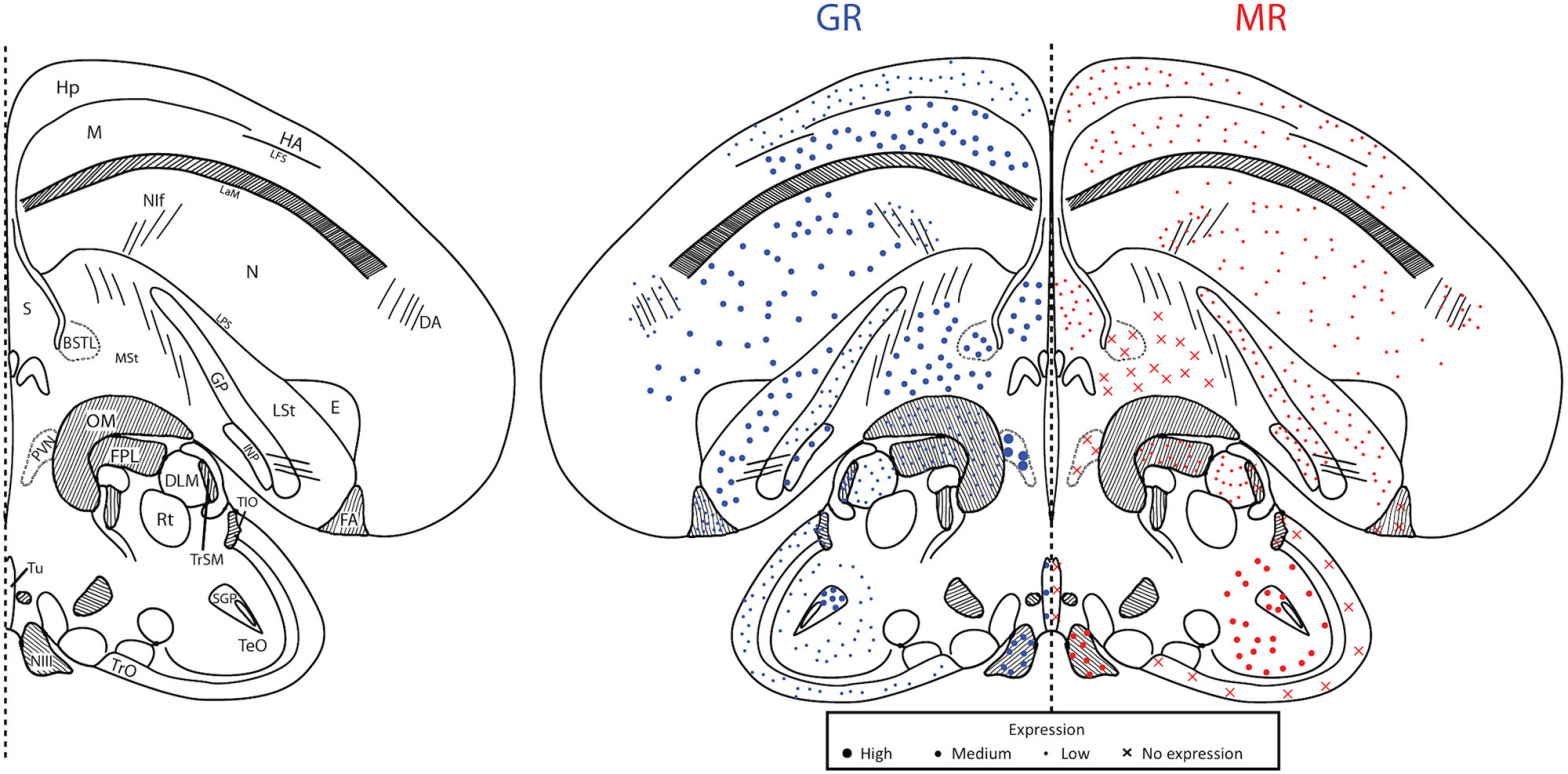
Plate 17 (A2.07–2.25 in the zebra finch atlas). Legend (left) and map (right). GR (blue, left) and MR (red, right) expression is indicated by dot size. An “X” indicates that the background expression met or exceeded the expression in the region. Area abbreviations can be found in Table 2.

**Fig 5:**
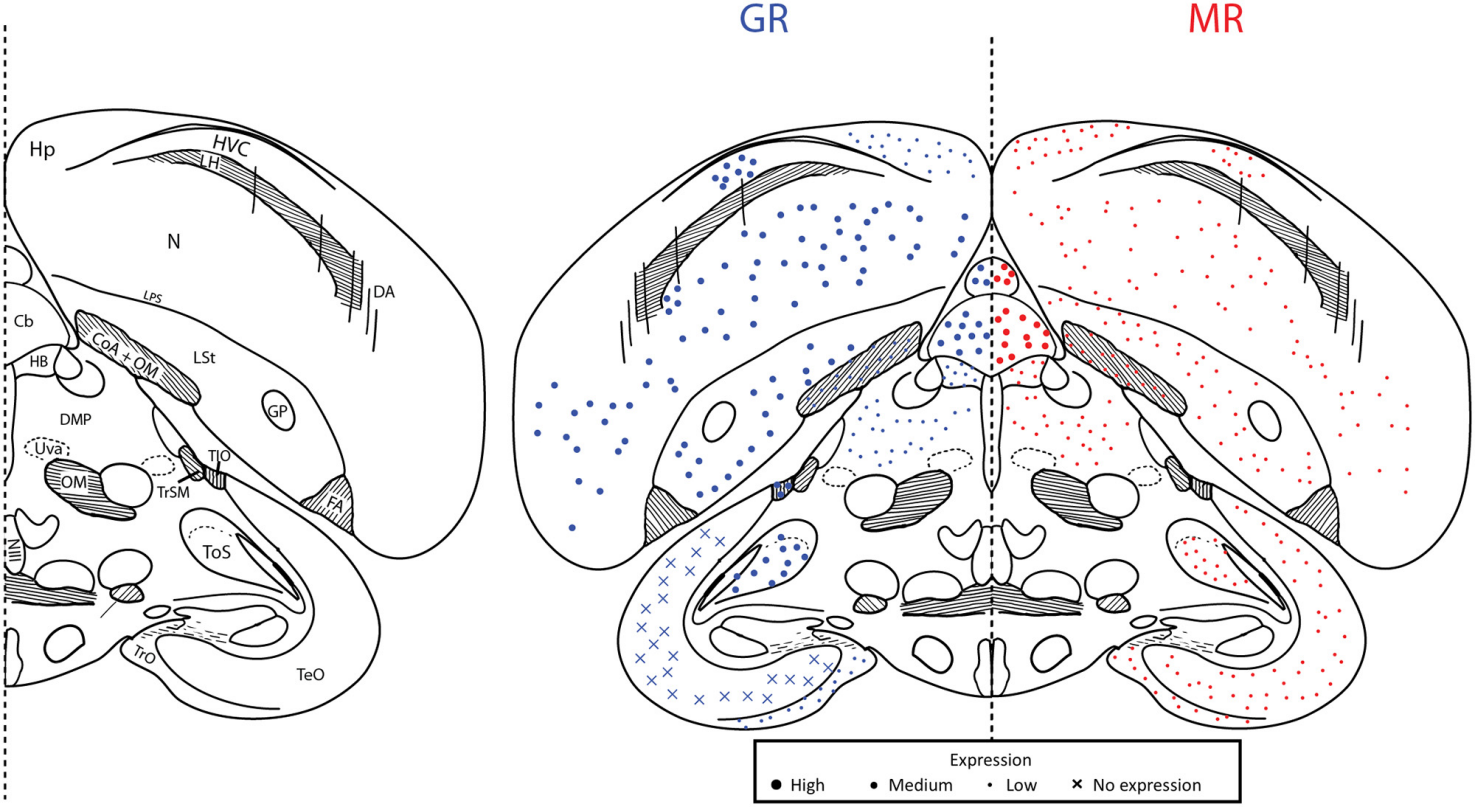
Plate 21 (A1.53–1.71 in the zebra finch atlas). Legend (left) and map (right). GR (blue, left) and MR (red, right) expression is indicated by dot size. An “X” indicates that the background expression met or exceeded the expression in the region. Area abbreviations can be found in Table 2.

**Fig 6:**
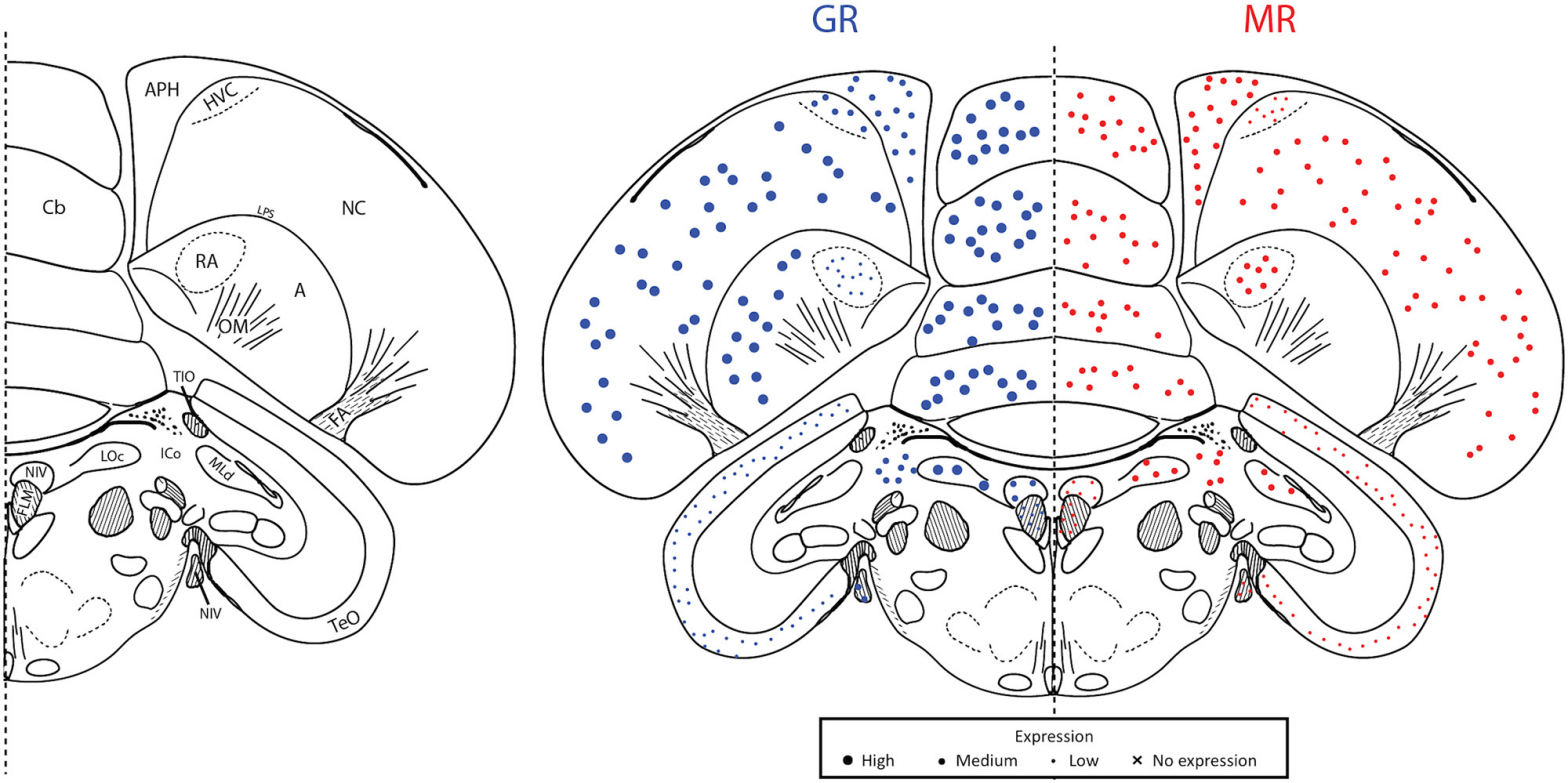
Plate 28 (A0.36 in the zebra finch atlas). Legend (left) and map (right). GR (blue, left) and MR (red, right) expression is indicated by dot size. An “X” indicates that the background expression met or exceeded the expression in the region. Area abbreviations can be found in Table 2.

**Fig 7:**
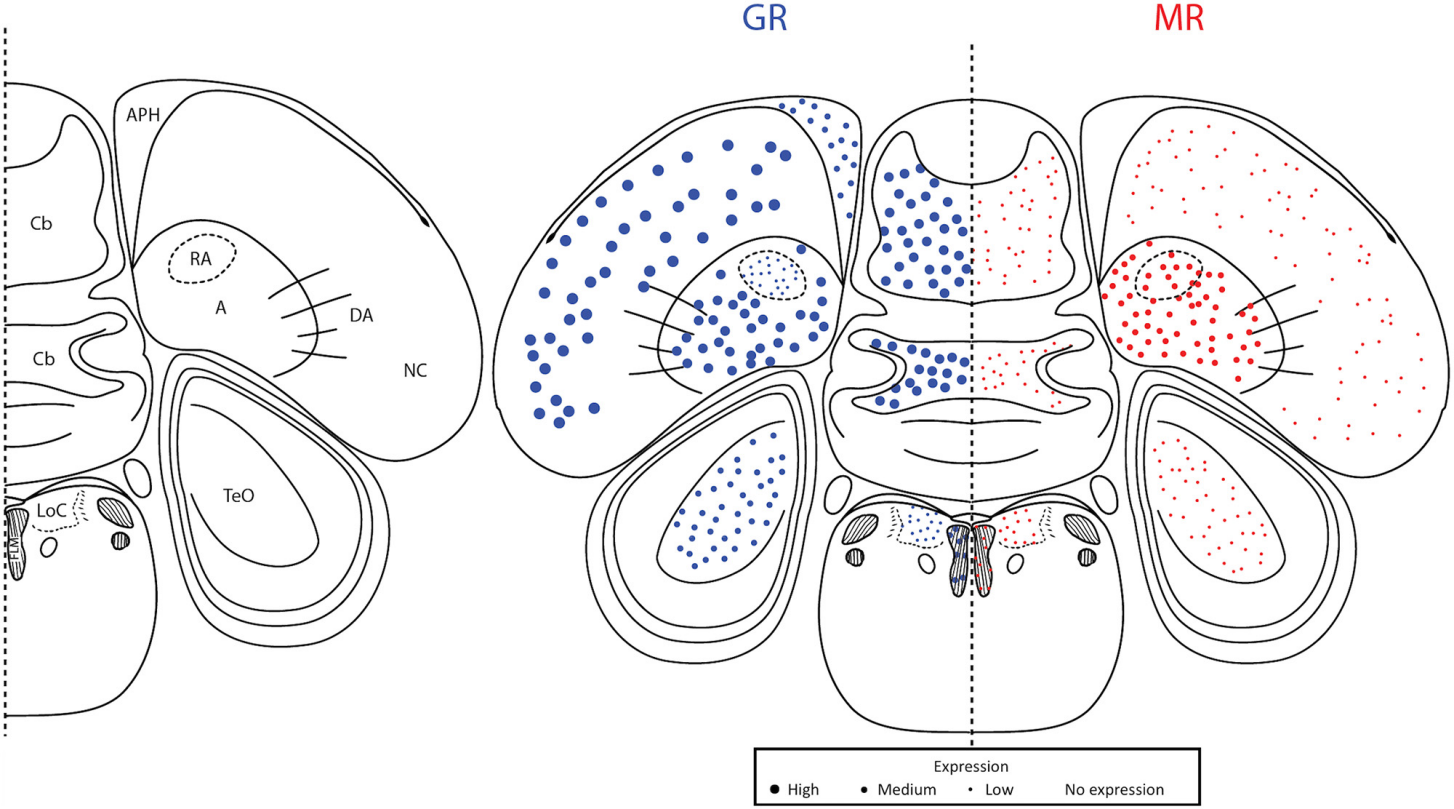
Plate 30 (A0.18–0.00 in the zebra finch atlas). Legend (left) and map (right). GR (blue, left) and MR (red, right) expression is indicated by dot size. An “X” indicates that the background expression met or exceeded the expression in the region. Area abbreviations can be found in Table 2.

**Fig 8:**
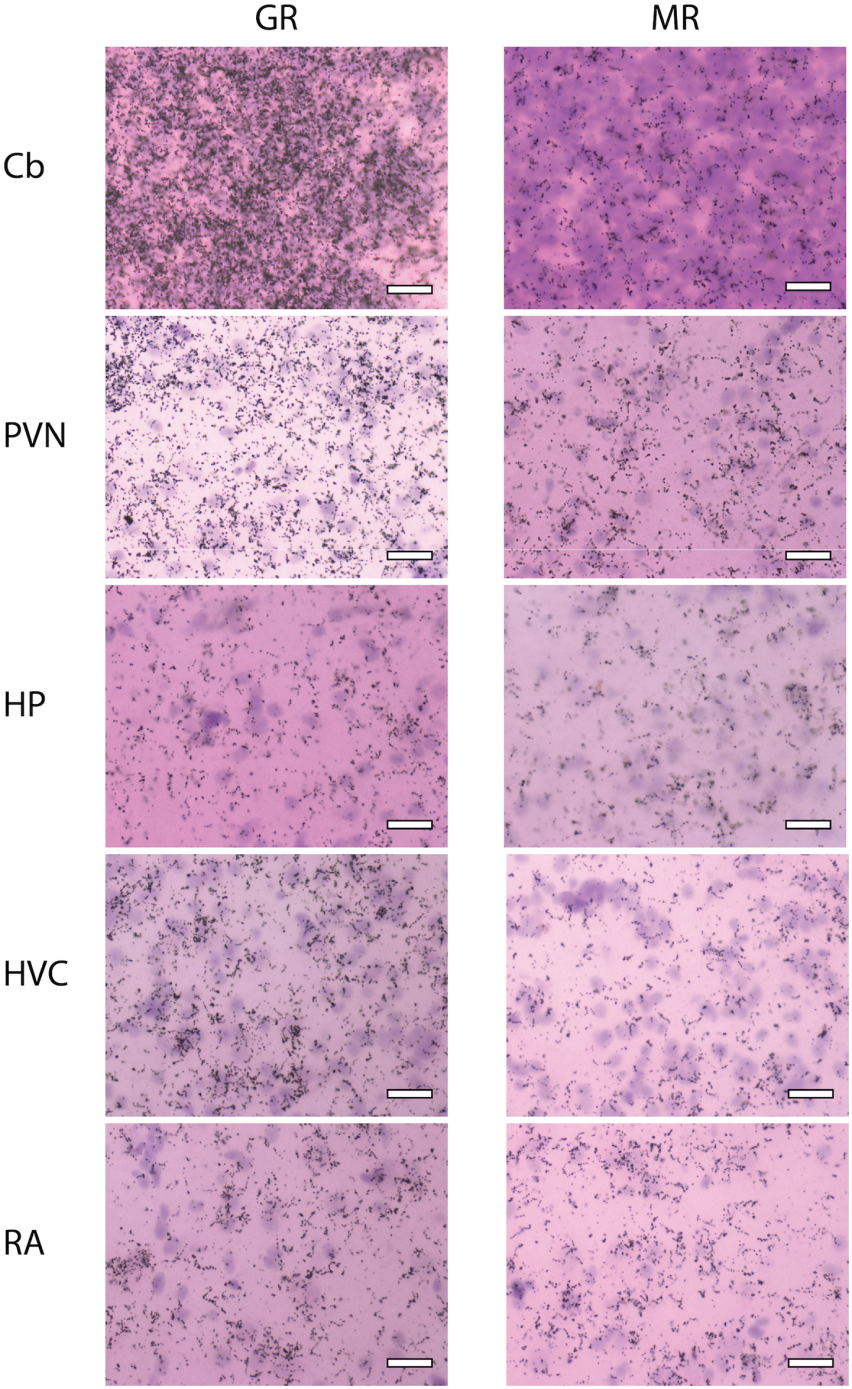
Representative photopictomicrographs of MR and GR expression. Cerebellum (Cb), paraventricular nucleus of the hypothalamus (PVN), hippocampus (HP), HVC, and nucleus robustus arcopallii (RA). Cell bodies are dark purple, silver grains are black dots. Scale bar indicates 50 μm.

Throughout the brain of a female great tit, we were able to sample MR and GR mRNA from 48 unique regions corresponding to six plates throughout the brain (Figs 2–7). These were roughly plates 6, 12, 17, 21, 28, and 30 in the stereotaxic canary atlas and the following ranges of plates in the zebra finch atlas: 10–11, 19, 25–26, 29–30, 35, 37–39 [34,35]. Because we sampled exhaustively from a single individual, it is necessary to be conservative in the generalizability of the receptor expression data. However, other distribution maps have used small numbers of individuals [37,38]. Additionally, we were able to sample HP and PVN across all 25 individuals and for these regions, the bird chosen for our distribution map is representative of the cohort (Table 1). For representative images of key regions, see Fig 8.

**Table 1:** Summary of average MR and GR expression in hippocampus (HP; n = 25) and paraventricular nucleus of the hypothalamus (PVN; n = 23) across all great tits and in the individual used for the distribution map (DM bird).

	GR			MR		
	DM bird $\bar{\text{x}}$	All birds $\bar{\text{x}}$	s.d.	DM bird $\bar{\text{x}}$	All birds $\bar{\text{x}}$	s.d.
**HP**	0.04085	0.05756	0.02756	0.06813	0.06207	0.03250
**PVN**	0.09574	0.1143	0.04668	0.03694	0.03105	0.02024

Data are untransformed, in units of pixels of silver grains over cell bodies minus pixels silver grains in background all divided by cell body area.

We found the highest GR mRNA expression, in descending order, in the cerebellum, locus coeruleus, nidopallium, apical and densocellular hyperpallium, and paraventricular nucleus of the hypothalamus. The highest MR, in descending order, was found in the nucleus septalis medialis, nucleus rotundus, cerebellum, trochlear nerve, and parahippocampal area. The lowest GR expression was found in the optic tectum, striatum laterale, and optic tract with no expression found in the optic tectum. For MR, low to no mRNA expression was found in the anterior commissure, optic tract, paraventricular nucleus of the hypothalamus, lateral part of the bed nucleus of the stria terminalis, and nucleus tuberis. These values describe lowest and highest sampled regions throughout the plates. For average expression across all plates, see Table 2.

**Table 2:** Summary of average GR and MR mRNA expression per region (−, +, ++, +++).

Abbreviation	Full name	GR	MR
**A**	Arcopallium	++	++
**APH**	Area parahippocampalis	++	++
**BSTL**	Lateral part of the bed nucleus of the stria terminalis	++	−
**Cb**	Cerebellum	+++	++
**CoA**	Commissura anterior	+	+
**CP**	Commissura posterior	+	NA
**DA**	Tractus dorso-arcopallialis	+	+
**DLM**	Nucleus dorsolateralis anterior thalami, pars medialis	+	+
**DMP**	Nucleus dorsomedialis posterior thalami	+	+
**DSV**	Decussatio supraoptica ventralis	+	++
**FA**	Tractus fronto-arcopallialis	++	+
**FLM**	Fasciculus longitudinalis medialis	+	+
**FPL**	Fasciculus prosencephali lateralis (lateral forebrain bundle)	+	+
**GP**	Globus pallidus	+	+
**HA**	Hyperpallium apicale	++	++
**HB**	Habenula	+	+
**HD**	Hyperpallium densocellulare	++	++
**Hp**	Hippocampal formation	+	+
**HVC**	formal name, located in nidopallium	++	+
**ICo**	Nucleus intercollicularis	+	++
**INP**	Nucleus intrapeduncularis	++	+
**LA**	Nucleus lateralis anterior thalami	+	NA
**LMAN**	Nucleus lateralis magnocellularis nidopallii anterioris	+	+
**LoC**	Locus coeruleus	++	+
**LSt**	Striatum laterale	+	++
**M**	Mesopallium	++	++
**MLd**	Nucleus mesencephalicus lateralis, pars dorsalis	NA	++
**MSt**	Striatum mediale	++	+
**N**	Nidopallium	++	++
**NC**	Nidopallium caudale	++	+
**NIf**	Nucleus Interfacialis nidopallii	+	+
**NIII**	Nervus oculomotorius	++	++
**NIV**	Nervus trochlearis	+	+
**OM**	Tractus occipito-mesencephalicus	+	NA
**PMH**	Nucleus medialis hypothalami posterioris	++	++
**PVN**	Nucleus paraventricularis of the hypothalamus	+++	+
**RA**	Nucleus robustus arcopallii	+	++
**Rt**	Nucleus rotundus	NA	+++
**S**	Nucleus septalis	++	+
**SGP**	Substantia grisea et fibrosa periventriculare	++	++
**SM**	Nucleus septalis medialis	++	+++
**TeO**	Tectum opticum	+	+
**TFM**	Tractus thalamo-frontalis et frontalis-thalamicus medialis	+	+
**TIO**	Tractus isthmo-opticus	+	+
**ToS**	Torus semicircularis	+	+
**TrO**	Tractus opticus	+	+
**TrSM**	Tractus septopalliomesencephalicus	+	+
**Tu**	Nucleus tuberis	++	−
**X**	Area X	+	+

In the present study, relative values (− no expression, + low expression, ++ medium expression, +++ high expression) were calculated by binning pixels of silver grains over cell bodies minus pixels silver grains in background all divided by cell body area.

### Song regions

We sampled MR and GR mRNA from five nuclei of the song system (1–4 sections per nuclei): HVC, NIf, LMAN, RA, and Area X. Regarding average expression, HVC expressed moderate GR and low MR; RA expressed low GR but moderate MR; and, LMAN, NIf, and Area X expressed low GR and low MR.

### Comparison across species

We compared our results to previous distribution maps of MR and GR in other songbird species: the European starling (*Sturnus vulgaris*), chukar (*Alectoris chukar*), zebra finch (*Taeniopygia guttata*), and Bengalese finch (*Lonchura striata domestica*) (Table 3). Because previous distribution maps in these species did not specify relative expression values, we have indicated whether expression was present (P) or absent (A) for those species. For *L*. *domestica*, male and female expression was assessed separately.

**Table 3:** MR and GR expression across species.

	Great tit (present study)		European Starling^1^		Alectoris chukar^2^		Zebra finch^3^		Bengalese finch (M)^4^		Bengalese finch (F)^4^	
Abbreviation	GR	MR	GR	MR	GR	MR	GR	MR	GR	MR	GR	MR
**BSTL**	++	−			P	A						
**Cb**	+++	++					P	A				
**DLM**	+	+							P	P	P	P
**HP**	+	+	P	P	P	P	P	P	P	P	P	P
**HVC**	++	+							P	P	*	*
**LMAN**	+	+							P	P	P	P
**N**	++	++					P	A				
**PVN**	+++	+	P	A	P	A	P	A				
**RA**	+	++							P	P	P	P
**TeO**	+	+	A	P								
**TrO**	+	+	P	A								
**Tu**	++	−	P	A								
**X**	+	+							P	P	*	*

In the present study, relative values (− no expression, + low expression, ++ medium expression, +++ high expression) were calculated by pixels of silver grains over cell bodies minus pixels silver grains in background all divided by cell body area. In other studies, presence (P) or absence (A). A blank cell indicates that region was not explored in that species. For abbreviations, see Table 2.

*This region was not found in the female Bengalese finch.

^1^Dickens et al., 2009,

^2^Dickens et al., 2011,

^3^Hodgson et al., 2007,

^4^Suzuki et al., 2011

Consistent with previous studies, we noted high expression of GR in the PVN and the presence of both MR and GR in HP. Unlike in these other species, however, we found relatively low MR in HP. Moreover, we found that MR and GR are present in the cerebellum optic tectum respectively, two regions that have not been reported previously to express these receptors; we note, however, that this apparent species difference could be a sex difference.

#### MR and GR expression in the PVN and HP across individuals

**Fig 9:**
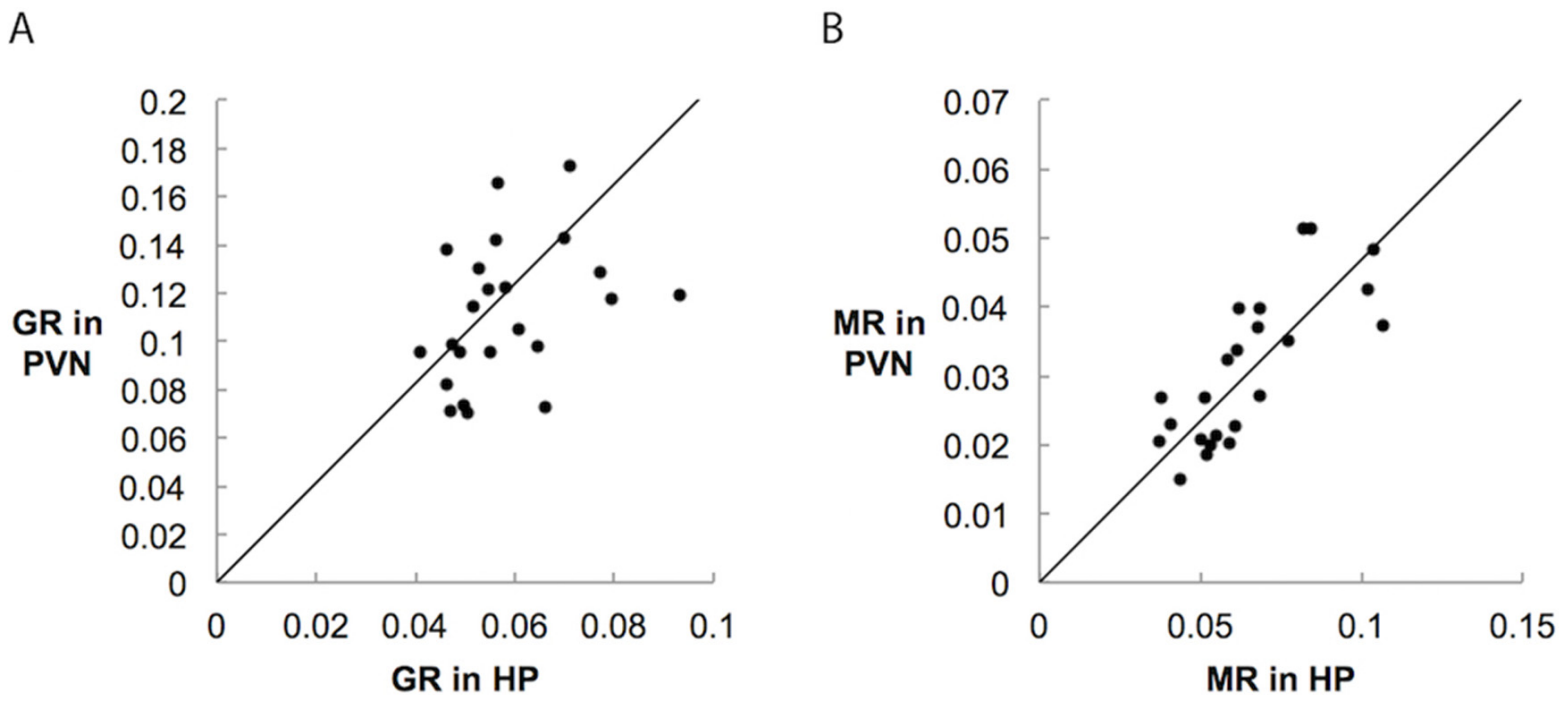
Expression of GR (A) and MR (B) regressions across HP and PVN. Units are area of silver grains superimposed over cell bodies minus background silver grain area divided by cell body area (see methods). Trendline and tests performed with log-transformed data, though untransformed data are shown. MR levels in HP significantly predicted MR levels in PVN (R^2^ = 0.5941, p<0.0001). GR levels in HP demonstrated a trend toward predicting GR levels in the PVN (R^2^ = 0.15, p = 0.069). No other combinations of areas and receptors were significant (all p>0.05).

For each of twenty-five birds, we assessed MR and GR expression in HP (n = 25) and PVN (n = 23). Average expression and standard deviations, as well as the values for the individual used in the distribution map, are reported in Table 1. There was no effect of sex on expression of MR or GR in either region (all Z<0.9, all p>0.37; Kruskal-Wallis test). We also created linear regressions to examine how receptor expression is correlated across and within regions. We tested all six combinations with MR or GR in the PVN or HP (Fig 9). Expression of MR across these two nuclei was strongly and significantly correlated (R^2^ = 0.59, F(1,21) = 30.73, β = 0.65, p<0.0001*; Fig 9A). Similarly, there was a trend towards GR expression being correlated across these two nuclei (R^2^ = 0.15, F(1,21) = 3.63, β = 0.30, p = 0.069; Fig 9B). All others were nonsignificant (all p>0.05) and are thus not shown in Fig 9.

## Discussion

The distribution and abundance of hormone receptors, even in model organisms, is poorly understood. This is in contrast the surplus of studies that have focused on circulating concentrations of hormone. Understanding which tissues, and in the case of the nervous system, which regions express hormone receptors provides a critical starting point for generating hypotheses about the endocrine and behavioral effects of the hormone of interest. Moreover, even closely-related species may have different patterns of receptor expression [13], which may help explain inter-specific variation in other traits and illustrates the general need for more studies that focus on receptors. Finally, understanding the extent to which steroid hormone receptor expression is coordinated across brain regions has the potential to reveal a mechanism facilitating the functional integration of multiple phenotypic traits (e.g. personality suites; [15–17]) and constrain phenotypic independence. For example, recent work has shown that the binding of glucocorticoids to GRs is necessary for maintaining a correlation between immune function and exploratory behavior [39]. Syncing receptor expression in different neural regions could allow glucocorticoid release to simultaneously affect multiple traits, resulting in the development of behavioral syndromes.

The distribution map reported here is one of the most anatomically comprehensive and resolved available for a songbird and is largely congruent with other songbird maps for these receptors. We found the highest GR mRNA expression in the cerebellum, where it is thought to play an important role in synthesis of leukotrienes (inflammatory agents found in immune cells) [40]. High expression levels have also been reported for other songbird species [10]. We also found high GR in the PVN, where it regulates HPA axis activity, and in the nidopallium, where its function has not yet been determined (Table 2). In contrast to a previous songbird map reporting no GR in the optic tectum [9], we found low, but detectable, expression in *P*. *major*.

MR was highest in the septalis medialis, nucleus rotundus, cerebellum, trochlear nerve, and parahippocampal area (Table 2). These regions were not examined in previous studies, though possible functions of MR can be hypothesized. For example, the septalis medialis is reported to be involved in sleep, particularly in the production of theta waves and slow wave sleep in mammals [41]. Because previous research has demonstrated homology between these regions in mammals and birds [42], MR expression could potentially play a role in regulation of sleep-wake states [43,44].

We also detected moderate MR mRNA expression in the cerebellum, and it is possible that MR may also play a similar role to GR in the cerebellum in regulating leukotriene synthesis, though future work will need to examine other species and better characterize MR function by identifying the specific DNA response elements to which MR binds.

Surprisingly, we found low MR expression in HP. This finding contrasts with a number of other songbird distribution maps [8–10], and may reflect inter-specific variation. Distribution maps on members of the primate family have found differences in mRNA levels in HP and other regions among closely-related species such as macaques and squirrel monkeys [13]. Again, such interspecific variation highlights the importance of creating distribution maps for each focal species.

Across our sample of birds, we found a strong positive correlation between MR expression in the PVN and HP. Expression of GR showed a similar, though nonsignificant trend between these two regions. This extends previous work [30], which examined receptor expression across many tissues throughout the body, by looking at specific regions of the brain known to be critical in regulating the HPA axis. Unlike this previous study [30], which found relatively weak correlations among different regions of the body, we found a strong correlation among brain regions. Birds with high MR in PVN also had high MR in HP. Such correlations might derive from these brain regions sharing a similar developmental program to each other compared with more disparate regions throughout the body [45]. Additionally, similar expression patterns could result from the anatomical and functional connectivity between the HP and PVN, as HP input to the PVN regulates the stress response by reducing its duration [46]. Future work should explore other regions identified by the distribution map here to explore how widespread such correlations are, and to assess if one of the receptors (e.g. MR) is generally more coordinated than the other. Such finding would provide support for the idea that neuroendocrine mediated hormonal pleiotropy integrates and coordinates phenotypic traits [24–29].

### MR and GR in the female song system

The song system in females is greatly underexplored compared to males [10]. It is a common assumption that female songbirds do not often sing in the wild and little work is done to explore the mechanisms and function of female song. However, in some songbirds, such as white-crowned sparrows, females do sing and this event is not rare in the wild [47]. A recent study of over 300 songbird species estimated that females sing in 71% of the studied species [48]. In addition, the authors showed that not only is female song common, it is the ancestral character state. Although great tits and *Passerida* were excluded from the study, female great tits are known to vocally interact with males during dawn chorus [49].

In addition to a potential role in production of female songs or calls, the song system also appears to be important in song perception. Intact female great tits can discriminate among conspecifics by song alone [50,51]. Lesioning of song system regions (e.g. HVC), however, renders female songbirds unable to discriminate between heterospecific and conspecific song, or between two different conspecific songs [52,53]. Further, the number of neurons and the volume of song system nuclei correlated positively with the selectivity of copulation solicitation displays in female brown-headed cowbirds (*Molothrus ater*) [54]. The discriminatory abilities conferred by the song system are also essential for maintaining social systems. In *M*. *ater*, inactivation of the song system led to decreased preference for high quality conspecific song, causing weaker pair bonds and in turn greater instability of the entire cowbird social network [55].

At present, a few studies have explored the effects of stress on female song recognition and discrimination [56–58], with effects that appear to vary depending on the type of stressor. For example, an early-life unpredictable food stressor reduced female preference for conspecific song in European starlings [57]. Additionally, the mechanism by which stress modulates these behavioral (and associated physiological) changes remains unknown. Our results suggest, however, that this would be a fruitful avenue for future research given the strong presence of GR in HVC and MR in RA. One important next step is to describe the developmental timelines of receptor expression [59], in conjunction with circulating concentrations of glucocorticoids [60]. The influence of early life stress (e.g., nutritional stress; [61]) and sex differences on developmental programming of the HPA axis could be examined in the context of long term effects on vocal learning and auditory perception.

We found that most song nuclei expressed low levels of MR and GR with the exception of HVC, which expressed moderate GR and RA, which expressed moderate levels of MR. These findings suggest that these two regions may be particularly sensitive to circulating glucocorticoids. Specifically, HVC may be sensitive to the high concentrations that accompany the stress response, potentially impacting song memory and discrimination abilities in females under stress [56–58,61]. Moderate levels of MR in the RA suggest that cells in this region may be particularly sensitive to glucocorticoids at low concentrations, as MR has a higher affinity than GR and is saturated at lower levels. Neurons from RA project onto motor neurons that then control the production of song by constricting the syringeal muscles in birds. Our distribution map suggests that stress-induced increases in glucocorticoids might impact neural activity thereby modulating song production. However, in both RA and HVC, the specific effects of activating MR or GR will depend on which response elements they target in these regions. Future work should aim to identify these response elements and their functional impacts.

In a study on male and female Bengalese finches, Suzuki et al. demonstrated dispersed, nonspecific GR expression and strongly clustered, song region-specific MR expression [14]. This is in contrast to the low receptor expression that we observed in the song system of great tits, which might indicate that these regions are relatively insensitive to changes in glucocorticoid concentrations. We recognize, however, that comparing among studies is challenging because receptor expression might exhibit seasonal variation. The size of song nuclei and glucocorticoid receptor expression, for example, are both known to vary seasonally, with the nadir of expression and song nuclei size both occurring in winter [62,63]. Brain tissue in our study was collected in late fall. It is largely unknown whether expression levels remain constant, either relatively or absolutely, among regions throughout the year or if there are region-specific seasonal variation. In house sparrows, MR expression in HP fluctuates seasonally, as did the relationship between MR and GR expression and secretion of CORT in response to a stressor [64]. This work suggests environmental factors may modulate the stress response through mechanisms other than adjusting MR and GR expression. The importance of such seasonal patterns in receptor expression and in the relationship between MR/GR expression and glucocorticoid secretion requires further comparative study. Lastly, future work should also aim to quantify protein levels [65], in order to examine the time-course and stability of receptor expression.

## Conclusions

In the present study, we report the first distribution map of neural MR and GR RNA expression in great tits. We detected high expression of GR mRNA in the PVN and low MR mRNA in the HP. In the female song system, we found moderate GR in HVC, and moderate MR in RA. We explored covariation of MR and GR levels in the HP and PVN. We found a strong, positive relationship for MR in these two regions and a similar trend for GR, extending previous research on inter-tissue patterns of glucocorticoid receptor expression [30]. The present work expands our understanding of which brain regions may be sensitive to circulating glucocorticoids and serves as a basis for future studies exploring how stress sensitivity may influence behavior, including song production and perception.
